# Sex differences in family planning knowledge, attitudes, and use in Uganda

**DOI:** 10.1186/s40834-021-00166-5

**Published:** 2021-08-01

**Authors:** Afra Nuwasiima, Agnes Watsemba, Allan Eyapu, Peter Kaddu, Justin Loiseau

**Affiliations:** 1 Living Goods, Kampala, Uganda; 2 Global Research & Evidence Strategy Living Goods, Nairobi, Kenya

**Keywords:** Family planning, sex, knowledge, attitudes, use, community health, Living Goods, Uganda

## Abstract

**Background:**

Literature is satiated with studies focusing on knowledge, attitude, and practices of family planning (FP) among the female population, conversely, the gaps in sex-disaggregated data on FP continue to exist. This study sought to report sex differences existing in FP knowledge, attitude, and use in Uganda.

**Methods:**

This study uses data from a household survey that covered 16 districts in Uganda. Multi-stage cluster randomized sampling was employed for participant selection. Bivariate analysis for categorical data was conducted. Multilevel logistic regression model was applied to model the effects of socio-demographic characteristics on the use of modern FP methods.

**Results:**

Data from 4,352 respondents in the ratios of 70 % females and 30 % of males were analyzed. The mean age was 28.7 SD (8.5) and was not significantly different between males and females. More male respondents had secondary or higher level of education (44 %) than females (36 %). Knowledge of at least one modern FP method was high, but small significant differences were revealed between males (96 %) and females (98 %). Significant knowledge differences were seen in specific FP methods. A higher proportion of females (71 %) than males (67 %) perceived modern FP methods as always available in the community whereas more males (40 %) believed that modern FP methods can result in infertility than females (35 %). There was high self-efficacy about family planning methods use in both males and females. The proportion of married females that reported using or their partner using a modern FP method was 39 % compared to 45 % reported by the married males. Approx. 53 % of the males compared to 37 % of the females that reported condom use also cited STI/HIV prevention as the main reason for condom use suggesting dual protection as a driver for use. Males, young adults, the more educated, and those in marriage or active relationships were more likely to use modern FP methods.

**Conclusions:**

Our study found significant sex differences in knowledge, attitudes, and use of FP methods. The young adults and more educated respondents were more likely to use FP methods. The high self-efficacy observed for both males and females is a signal that both sexes can use FP methods. Project strategies and implementation should take into consideration the existing differences by sex and devise sex-tailored approaches to improve FP knowledge, attitudes, and use in this population. There was increased reporting of condom use as an FP and STI/HIV prevention method, follow-up studies aiming at succinctly measuring dual protection, and its drivers for both sex should be done.

## Background

Use of family planning (FP) methods by women and men can improve the health, economic, and social domains of their lives [1]. As such, FP continues to be a key focus of the global agenda for improving maternal, sexual and reproductive health. According to the Uganda Demographic and Health Survey (UDHS) report 2016, some knowledge of FP methods is nearly universal in Uganda, with 99 % of both women and men having heard of at least one method of FP [2]. However, the use of modern FP and any form of FP among married women in Uganda stood at only 35 and 39 % respectively. FP methods use among sexually active unmarried women was slightly higher with 51 % using any FP method and 47 % using a modern method, and the total unmet need for FP among the sexually active women of reproductive age stood at 28 % among currently married women, and 32 % among the sexually active unmarried women.

In the FP Costed Implementation Plan 2015–2020 [3], the Uganda Ministry of Health (MoH) acknowledged that several innovative FP service delivery models have been successfully implemented by Non-Governmental Organizations (NGOs) in Uganda in an effort to remove access barriers for FP. For instance, community-based distribution of certain FP commodities has been successful at expanding access, and task shifting of injectable FP to Village Health Teams (VHTs)/Community Health Workers (CHWs). Over the past 2 decades, government-supported VHTs have provided a broad service package including family planning. However, key challenges still exist, limiting the effectiveness of the community health program; CHWs not adequately equipped with commodities and skills to deliver FP, absence of supervision, no compensation, and suboptimal use of reporting and decision support tools. In 2018, Living Goods, an international organisation piloted the implementation of comprehensive CHW led FP services model while addressing these gaps in the two districts of Wakiso and Mpigi in Uganda. The FP services provided to the clients mainly comprised of counseling, and provision of condoms, DMPA-SC (commonly known by the brand Sayana Press® or simply Sayana) and oral FP and as well as referral for long-term methods. The pilot program results suggested that by working through CHWs, it is possible to address the binding constraints to the uptake and utilization of FP including social opposition, misinformation and fear of side effects, among others[4].

Men’s knowledge, attitudes, and behaviors around FP impact uptake and unmet need because men are not only women’s partners, but individuals with distinct reproductive histories and desires of their own. A 2020 Tanzania study [5] found that knowledge of modern contraception was a crucial factor in influencing the use of modern FP among men and suggested that targeting of men with FP awareness programs would improve the use of FP among men. The UDHS 2016 findings [2] showed that 12 % of married women were refused to use FP by their partners while 44 % made the decision jointly with their husbands. The literature on the role and attitude of men in decision-making is mixed. A study in Uganda revealed that men were greatly interested in modern contraception, albeit they possessed little knowledge about it [6] while a related study in Tanzania showed that men expressed little interest in the use of modern FP methods, and yet were considered by women as key decision-makers in their use of FP[7]. Another study conducted in Uganda using nationally representative data revealed that the use of FP was significantly higher among men that had discussions with a health worker, desired for few children, belonged to a higher wealth index, and highly educated [8], and these factors are similar to factors that affect FP among women. Male inclusion in FP has been encouraged by various international and national initiatives including providing incentives for men who escort their partners for FP. However, gaps in data continue as FP data in programs and reports is rarely disaggregated by sex if men are included at all [9]. Where data on utilization of modern contraception were reported among men, FP methods use was found to be high[5].

Whereas there is paucity of literature on men’s statistics, collection, analysis, and reporting of sex-disaggregated data are critical to fully understand the specific needs of men and women. This study sought to explore sex variations existing in the knowledge, attitudes, and use of FP services in selected districts of Uganda.

## Methods

### Study design

This was a cross-sectional survey, conducted as a baseline to a larger research project. The paper focuses on the results from the quantitative household survey. Data collection was conducted in August 2019.

### The study setting

**Fig. 1:**
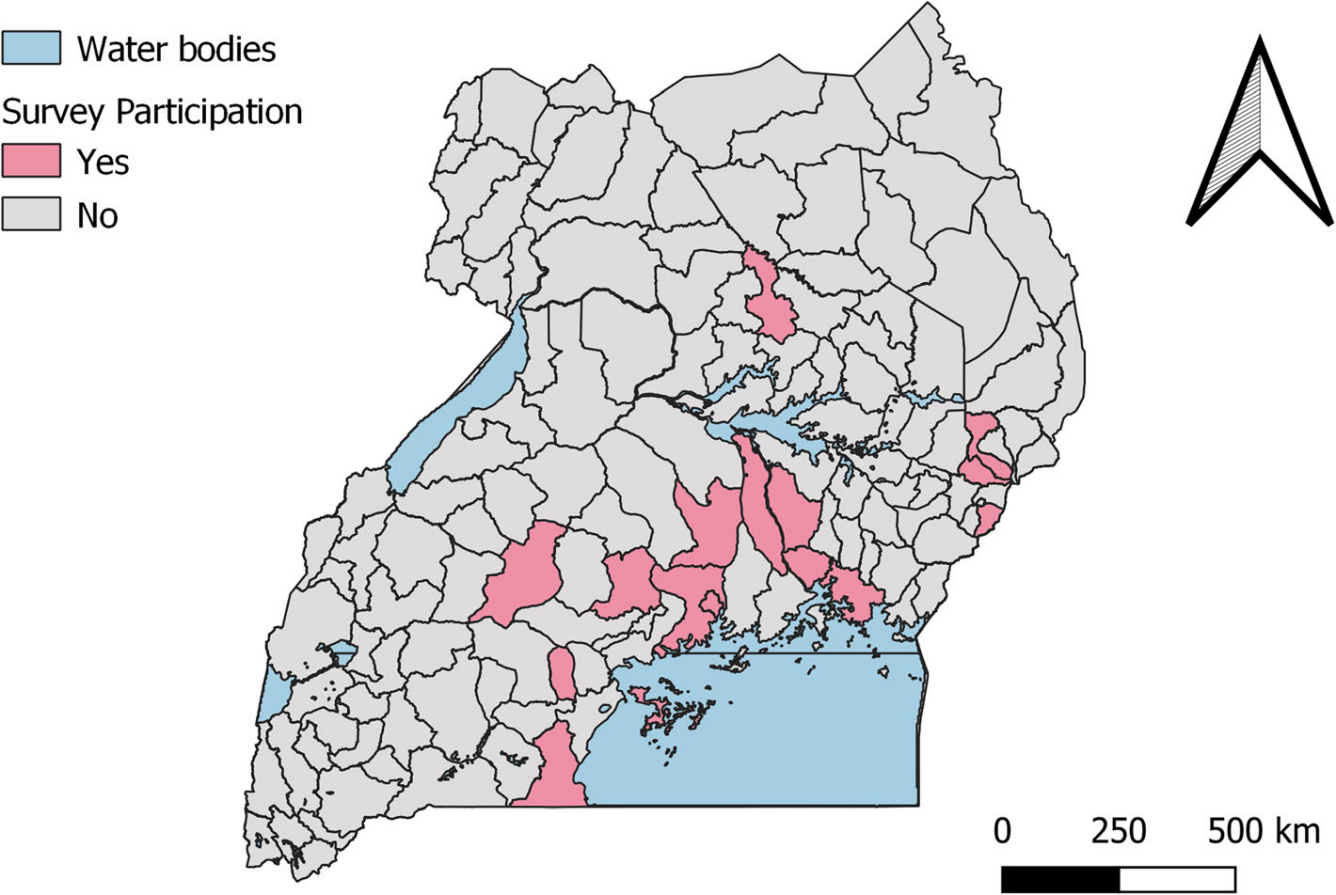
Map showing the survey districts in Uganda

Data collection was undertaken in sixteen (xvi) districts of Uganda. The study did not seek to be nationally representative as the selection of study districts took into consideration Living Goods Uganda’s current catchment area for the FP intervention. Figure 1 shows a map of Uganda with survey districts marked “Yes” with a pink color.

### Study population

Participants in the household (HH) baseline survey comprised of women and men in the reproductive age of 15 to 49 years.

### Sample size

The Cochran (1963) formula for two independent samples with a dichotomous outcome was used to determine the sample size (n). To achieve a maximum sample size, a proportion of 0.5 per sample group was used. A sample of 4,380 respondents was derived with a statistical power of 90 %, a precision level of 5 %, a design effect is 1.5, and a 95 % confidence level. The sample was distributed equally among sampled districts and villages.

### Household sample selection

The selection of the households for the survey utilized a four-stage sampling approach. The first stage involved a purposive selection of districts for the survey and the second stage involved a random selection of sub-counties per district (2 to 6 sub-counties per district). The third stage involved the random selection of four villages within each sub-county using the computer-based Random Draw Method. The fourth and last stage involved systematic random sampling of households to be interviewed. To ensure all eligible households had an equal chance of participating in the survey, the survey team worked with local leaders to conduct a household listing of households with persons in the age range of 15–49 years (male and female). The desired ratio of 3 females to 1 male was used. The sample size was not adjusted for non-response and consequently, the household replacement was assumed to be feasible where a HH refused to participate or was not available at the time of interview. For the latter non-response, the replacement was done after making three [3] call-backs to the household. For each HH selected, only one person was needed for an interview. Therefore, simple random sampling using the raffle/lottery method without replacement was used to select a respondent from all eligible members of the desired sex in the household. The sex of the respondent was predetermined before arriving at the household.

### Data collection methods

The quantitative household survey data collection was done through face-to-face interviews using a structured questionnaire. The questionnaire was programmed using the Open Data Kit (ODK) software and uploaded on electronic devices (smartphones).

### Quality assurance

Before the study was started, a data quality assurance protocol was developed and this included constituting a strong and dynamic team with experience in conducting similar assignments, recruiting and training experienced research assistants and field supervisors to handle data collection. The ODK data collection tool was also designed with controls to limit data entry errors. Field supervisors did spot checks on 5 % of the respondents to validate the information collected by the interviewers and feedback was analyzed to improve the process. Also, the research team organized routine internal meetings to review progress on the data collection against set targets per district per cluster.

### Data analysis

Descriptive analysis using proportions was conducted to show the distribution of FP knowledge, attitudes, and use of FP. Bivariate analysis using Pearson’s Chi-square and Fisher’s exact tests for categorical data was done. Additionally, a multilevel logistic regression model [10] was used to model the effects of socio-demographic characteristics on the use of family planning methods. The sex of the respondent was included in the model as a fixed factor to model sex differences. To model the associations of respondents within the different districts, sub-counties, and villages, the study considered a four-level analysis model with three high-level cluster variables (random effects equations) i.e. district, sub-county, and village treated as random effects factors to account for within-cluster correlations (intra-cluster correlation). This assumes that observations nested in a cluster share common characteristics in terms of access to community services and this may affect the use of family planning methods. All quantitative analysis was done in STATA version 15 software and statistical significance was considered at 5 %.

### Ethical considerations

 The study received ethical clearance from Mildmay Uganda Research and Ethics Committee (MUREC) and Uganda National Council for Science and Technology (UNCST). All members of the survey team were trained on the protection of human subjects. In the field, all members of the survey team obtained written informed consent, upheld the principles of voluntary participation, confidentiality, anonymity, and respect of the privacy of respondents, and the obligation to not do any harm. Identifiers such as names of respondents were not tagged on an interview recorded on the smartphones further improving the privacy and confidentiality of survey data.

## Results

The quantitative study achieved an overall response rate of 99 % (100 % for women and 99 % for men) by reaching 4,352 respondents out of the targeted 4,380 respondents. The study managed to achieve the ratios of 70 % female and 30 % male representation in the quantitative survey. All 4,352 respondents had complete data on socio-demographic characteristics and main outcomes hence no missing data handling approaches were used.

### Socio-demographic characteristics of respondents

Table 1 shows the distribution of the socio-demographic characteristics of the study participants disaggregated by sex. The distribution of respondents differed by sex on the following variables; age group, education level, marital status, the main source of income, and the number of children. Although there were observed significant differences in age groups for males and females starting at the age of 30, the overall mean age of respondents was 28.7 SD (8.5) and was not significantly different between males and females. Overall 39 % of the respondents had not achieved at least a primary leaving certificate with a higher proportion of females (41 %) than males (35 %). Approximately one-quarter of respondents were not married, with the percentage unmarried was higher among males (44 %) than females (21 %). A significantly lower percentage of females (16 %) than males (38 %) reported having no children.

**Table 1:** Socio-demographic characteristics of respondents by sex

Characteristic	Distribution			
	Overall n = 4,352	Females n = 3,061	Males n = 1,291	P value
Mean Age (SD)	28.7 (8.5)	28.7 (8.5)	28.5 (8.5)	0.915
**Age group (%)**				
*15–19*	14.2	13.9	14.9	0.000*
*20–24*	24.6	24.7	24.4	
*25–29*	19.2	19.4	18.7	
*30–34*	14.7	15.3	13.3	
*35–39*	12.8	13.7	10.6	
*40–44*	10.8	9.7	13.5	
*45–49*	3.6	3.3	4.5	
**Highest education level attained (%)**				
*None/ no education*	5.6	6.5	3.5	0.000*
*Primary incomplete*	33.7	34.8	31.1	
*Completed primary*	21.9	22.1	21.4	
*Secondary or higher*	38.8	36.6	44.1	
**Religious affiliation (%)**				
*Muslim*	18.7	18.2	20.1	0.208
*Roman Catholic*	36.7	36.5	37.1	
*Protestant*	31.9	32.5	30.5	
*Born again/Pentecostal*	10.2	10.5	9.2	
*None/Others*	2.6	2.3	3.1	
**Marital status (%)**				
*Single*	14.2	10.9	22.0	0.000*
*In a relationship*	12.8	10.5	18.2	
*Married / cohabiting*	64.3	68.3	54.9	
*Separated / divorced*	7.1	8.0	4.7	
*Widowed*	1.6	2.2	0.2	
**Residence of respondent (%)**				
*Urban*	29.1	28.7	29.8	0.593
*Trading Center*	12.7	12.5	13.2	
*Rural*	58.7	58.2	57.0	
**Main source of household income**				
*Crop farming/Cattle keeping*	47.1	46.4	48.9	0.007*
*Fishing*	1.8	1.7	2.1	
*Salary/Wage*	14.0	14.1	13.7	
*Business*	28.1	29.5	24.6	
*Boda-Boda / Other transport*	4.4	4.3	4.6	
*None*	0.5	0.5	0.7	
*Others*	4.1	3.6	5.3	
**Number of children (%)**				
*0*	22.9	16.4	38.3	0.000*
*1*	15.6	16.5	13.3	
*2*	14.2	16.0	9.9	
*3*	11.7	13.0	8.5	
*4*	9.8	10.7	7.7	
*5+*	25.7	27.2	22.1	

* Implies statistically significant results at 5 % level of significance

#### Knowledge of FP methods

Table 2 shows the results of the respondent’s knowledge of FP methods. Both sexually active and inactive respondents were included in the analysis. The findings revealed that overall 97 % of the respondents had heard of at least one modern FP method and there was a small, but significant difference between males (96 %) and females (98 %). There were also significant differences in knowledge of specific FP methods by sex. Women were most likely to know injectables, pills, implants, and IUDs. Male knowledge was highest for male condoms, followed by the same three methods as for females. Overall, the study observed higher levels of knowledge in hormonal birth control FP methods (implant, IUD, Injectable, Pills) than the natural methods (Lactational Amenorrhea, Withdrawal, Periodic abstinence) and permanent methods (female and male sterilization). Knowledge of male condoms was higher for both males and females than the female condoms.

**Table 2:** Knowledge of different FP services by sex of respondent

Measure	Distribution (%)			P-value
	Overall n = 4,352	Females n = 3,061	Males n = 1,291	
*Ever heard of any family planning method*	97.4	97.8	96.4	0.006*
*Ever heard of any modern family planning method*	97.2	97.6	96.2	0.012*
*Aware of all modern FP methods*	0.97	1.08	0.70	0.240
**Knowledge of specific FP methods**				
*Injectables*	83.6	89.5	69.5	0.000*
*Pills*	74.8	79.2	64.4	0.000*
*Implants*	66	73	49.3	0.000*
*Male condoms*	60.3	53.3	77.1	0.000*
*IUD*	53.9	60.6	38	0.000*
*Periodic abstinence*	16.6	18.1	13	0.000*
*Withdrawal*	15	14	17.2	0.008*
*Female condoms*	14.2	14.5	13.7	0.429
*Female sterilization*	13.5	14.7	10.7	0.000*
*Male sterilization*	11.7	9.9	16	0.000*
*Emergency FP pills*	9.2	9.8	7.7	0.024*
Lactational Amenorrhea	6.4	7.9	2.7	0.000*

* Implies statistically significant results at 5 % level of significance

### Use of contraception methods

Use of contraception was assessed by looking at the proportion of sexually active respondents that were not currently pregnant that reported using the different FP methods (84 % of the total respondents). Table 3 shows the proportion of respondents that reported the use of FP methods. The use of modern FP stood at 38.7 % overall and this was significantly higher among the males (45 %) than the females (36 %). The main methods of FP reported by females were Depo (37 %), withdrawal (22 %), and male condoms (9 %) compared to male condoms (46 %), Depo (19 %), and withdrawal (12 %) reported by males. Overall the use of the permanent FP methods was higher (6 %) than long-term methods (3 %). For permanent use, there were significant differences between males and females, but, no significant sex differences were observed among the long-term methods.

**Table 3:** Proportion of respondents reporting use of FP methods

FP methods use	Distribution (%)			P-value
	Overall (n = 3,677)	Females (n = 2,614)	Males (n = 1,063)	
*Currently using any FP method*	50.3	48.6	54.7	0.001*
*Currently using a modern FP method*	38.7	36.0	45.3	0.000*
**Main FP method used**				
***Short-term***				
Pills	4.5	4.7	4.1	0.558
Injectable (Depo)	31.1	36.5	19.1	0.000*
Injectable (Sayana Press)	5.9	7.7	2.0	0.000*
Emergency FP	0.7	1.0	0.0	0.013*
Male condoms	20.3	8.6	45.7	0.000*
Female condoms	0.1	0.1	0.0	1.000
***Long-term***				
IUD	2.6	2.9	1.9	0.196
Implants	0.2	0.1	0.5	0.095
***Permanent***				
Male sterilization	1.6	2.0	0.7	0.029*
Female sterilization	4.0	4.8	2.0	0.004*
***Natural***				
Lactational Amenorrhea	5.5	5.2	6.1	0.393
Periodic abstinence	4.1	4.0	4.3	0.785
Withdrawal	18.8	21.7	12.4	0.000*
***Others***	0.7	0.5	1.2	0.134

* Implies statistically significant results at 5 % level of significance

**Fig. 2:**
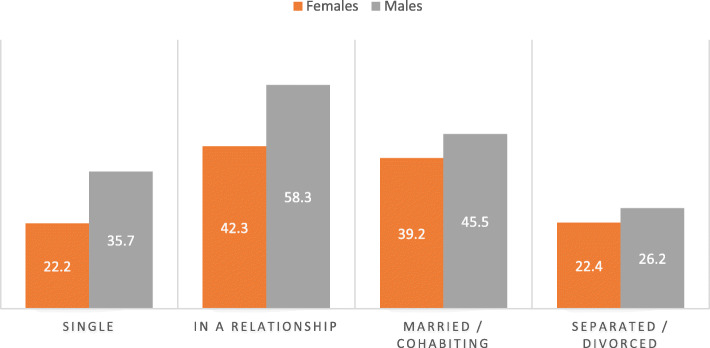
Modern FP use by marital status and sex

Figure 2 shows the use of modern FP methods among women by marital status and sex. The findings indicated that the proportion of married/cohabiting females that reported using or their partner using a modern FP was 39 % lower than 45 % reported by the male counterparts. Modern FP use was highest for both males and females that were in a relationship but married/ cohabiting and lowest for both males and females that were single or separated.

### Condom use as a dual protection method

**Fig. 3:**
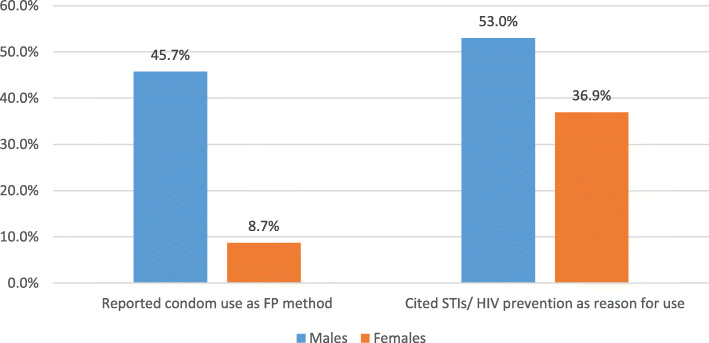
Proportion of respondents that reported condoms use as an FP method and a form of protection against STIs/HIV infection (males = 268 and females = 111)

Figure 3 shows that half of the males and a third of the female respondents that reported condom use as the current method of FP used it as a prevention measure of STI/HIV infection. The study did not establish whether the primary objective was STI/HIV protection or protection against pregnancy or both, and this is a limitation to accurately measure the differentiated effect of condom use on the results reported among males.

#### Respondent perceptions, beliefs, and self-efficacy about FP services

Table 4 shows the respondent’s perceptions on the availability, affordability, self-efficacy, and beliefs about the FP methods disaggregated by sex. The results in this section were restricted to men (92 %) and women (96 %) respondents that were in-union or sexually active singles. The results are explained in the following sub-sections.

**Table 4:** Perceptions of community members on FP services by sex

Perception/Attitude		Overall (n = 4,128)	Female (n = 2,936)	Male (n = 1,192)		P-value
**Perceived availability of FP services in the community**						
• *Agree that modern FP are always available in my community when I need them*		69.9	71.1	66.7		0.005*
**Perceived affordability of FP services in the community**						
*Modern contraceptives are affordable in my community*		52.6	52.3	53.5		0.457
**Community perception of the quality of FP services**						
• *Agree that FP services provided in public health facilities are of high quality*		73.1	75.0	68.5		0.000*
**Beliefs about FP services**						
• *Believe that using modern FP can result in infertility*		36.4	35.1	39.7		0.005*
• *Believe that using IUDs can result in cancer*		41.9	41.6	42.5		0.590
• *Believe that using Sayana Press reduces sexual pleasure*		11.4	12.5	8.6		0.000*
**Self-efficacy about FP services**						
• *I am embarrassed to get/ask about FP from a health facility*		11.3	11.2	11.5		0.816
• *I would be embarrassed if people found out that I am using FP*		10.0	9.9	10.0		0.790
• *It is ok for a woman/girl to suggest to her male partner that they use a condom or another method to avoid pregnancy*		87.5	87.9	86.4		0.198
• *My partner would be annoyed with me if they discovered I was asking for condoms, pills or other FP methods*		29.8	34.2	18.7		0.000*
• *My friends would laugh at me/ tease me if they found out that I was asking for condoms, pills or other FP services*		12.6	13.9	12.1		0.108

* Implies statistically significant results at 5 % level of significance

#### Perceived availability of FP services

Overall, more than half of the respondents (69.9 %) perceived modern FP as always available in the community whenever they were needed, and this was significantly higher among the females (71.1 %) than the males (66.7 %).

#### Perceived affordability of FP Services

The results in Table 4 further suggest that over half of the respondents (53 %) perceived modern FP methods to be affordable within their communities. There were no significant sex differences.

#### Perceived quality of FP services

The study results also revealed that overall almost three-quarters (73 %) of the respondents perceived public health facilities to be providing high-quality FP services, and this was significantly higher among the females (75.0 %) than males (68.5 %).

#### Beliefs about FP services

Beliefs about FP were assessed by exploring the respondent’s perceptions about the side-effects of FP considering; beliefs about modern FP and infertility, cancer, and the effects of FP use on sexual pleasure. Table 4 shows that more males (40 %) significantly perceived modern FP to result in infertility than females (35 %). For specific methods, overall 42 % of the respondents believed that the use of IUDs can result in cancer, and no significant sex differences were revealed. Overall, also, 11 % of the respondents believed that using Sayana Press reduces sexual pleasure, and this was lower among males (8.6 %) than females (12.5 %).

### Perceived role of CHWs in FP service delivery

**Fig. 4:**
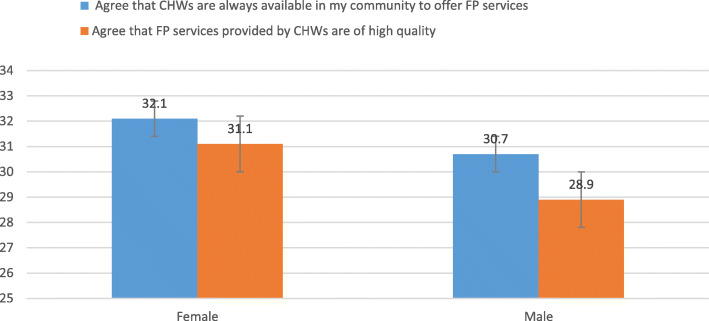
Perceived role of CHWs in FP service delivery

The study sought to establish the sex-disaggregated perception of the involvement of CHWs in FP services provision in communities. The results are presented in Fig. 4. Overall, there was perceived low involvement and quality of CHWs in FP services. Approximately only 3 in 10 of the respondents agreed that CHWs were available in the community to offer FP services and agreed that FP services provided were of high quality. There were no significant sex differences observed.

### Decision-making regarding FP use

**Fig. 5:**
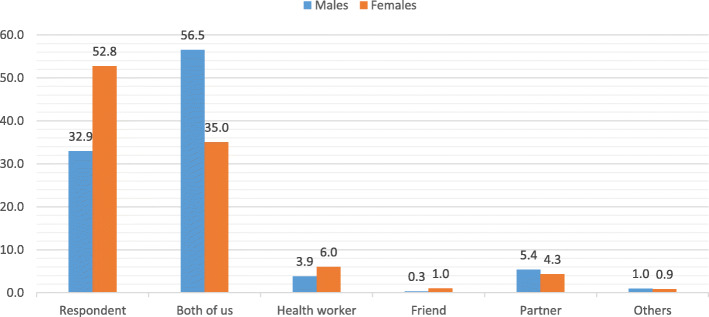
Decision making in FP uptake

Decision-making regarding FP use is a critical factor in the adoption of FP services. The results presented in Fig. 5 suggested that a larger proportion of females (53 %) decided for the couple to take on FP than 33 % reported by males. More males reported that the decision for the couple to use FP was joint compared to females. Even though half of the women reported deciding to use FP themselves, majority of them indicated that their partners were aware of the FP use.

### Effect of socio-demographic characteristics on modern FP use

Table 5 shows findings from the adjusted multilevel logistic regression to assess the effect of socio-demographic characteristics on modern FP use. The findings indicated that females were 35 % less likely to use FP compared to males (Adjusted odds ratio (AOR) = 0.65, P = 0.000). Respondents aged 40–44 and 45–49 were 36 and 69 % less likely to use modern FP compared to those aged 15–19. No significant differences were observed for other age groups in comparison to the 15–19 age group. For education level, those that completed primary were 43 % more likely to use modern FP (AOR = 1.43, P = 0.041) compared to those with no education while those that achieved secondary or higher were 45 % more likely to use modern FP compared to those with no education (AOR = 1.45, P = 0.030). After adjusting for sex and other socio-demographic characteristics, those in a relationship were approximately two times (AOR = 2.48, P = 0.000) more likely to use FP compared to those in no/ single relationships. The married respondents were also 88 % more likely to use modern FP compared to those in no relationship. No significant differences were revealed for religion, place of residence, source of income, and the number of children.

**Table 5:** Multilevel multiple logistic regression model findings on modern FP use

Characteristic	Modern FP use	
	AOR (95 % CI)	P value
**Sex of respondent**: *Ref = Male*		
Female	0.65 (0.55,0.76)	0.000*
**Age of respondent**: *Ref = 15–19*		
*20–24*	0.98(0.75,1.29)	0.908
*25–29*	1.12(0.83,1.51)	0.469
*30–34*	1.01(0.721.42)	0.960
*35–39*	0.88(0.61,1.27)	0.504
*40–44*	0.64(0.44,0.95)	0.028*
*45–49*	0.39(0.23,0.66)	0.000*
**Highest education level**: *Ref = None/ no education*		
*Primary incomplete*	1.21(0.87,1.69)	0.253
*Completed primary*	1.43(1.01,2.03)	0.041*
*Secondary or higher*	1.45(1.04,2.04)	0.030*
***Religion***: Ref = Muslim		
*Roman Catholic*	1.01(0.82,1.24)	0.894
*Protestant*	0.90(0.73,1.11)	0.345
*Born again/Pentecostal*	0.79(0.60,1.05)	0.108
*None/Others*	0.80(0.38,1.66)	0.545
**Marital status**: Ref = *Single*		
*In a relationship*	2.48(1.85,3.32)	0.000*
*Married / cohabiting*	1.88(1.39,2.54)	0.000*
*Separated / divorced*	0.85(0.57,1.27)	0.434
*Widowed*	0.64(0.30,1.37)	0.250
**Residence**: *Ref = Urban*		
*Trading Center*	0.92(0.70,1.20)	0.519
*Rural*	1.06(0.87,1.30)	0.552
**Household main source of income**: *Ref = Crop farming/Cattle keeping*		
*Fishing*	0.90(0.52,1.56)	0.711
*Salary/Wage*	1.05(0.83,1.31)	0.690
*Business*	0.99(0.83,1.18)	0.918
*Boda Boda/ Other transport*	1.13(0.79,1.60)	0.501
*None*	2.54(0.95,6.80)	0.062
*Others*	0.95(0.66,1.37)	0.791
**Number of children**: *Ref = No child*		
*1*	0.84(0.64,1.12)	0.237
*2*	1.30(0.95,1.78)	0.098
*3*	1.31(0.93,1.84)	0.119
*4*	1.33(0.91,1.92)	0.137
*5+*	1.23(0.86,1.86)	0.252

* Implies statistically significant results at 5 % level of significance

## Discussion

Literature is replete with studies focusing on knowledge, attitude, and use of FP services among the female population, conversely, the disaggregation of results by males and females has been less of a focus and not emphasized in reporting [9]. This paper’s findings add to a growing body of literature on sex-disaggregated FP knowledge and use. The results from the study revealed significant sex differences in the knowledge, attitudes, and use of FP services in selected districts in Uganda.

The finding that knowledge of FP was equally high for both males and females, despite the small differences is an indication that males and females have been equally targeted and engaged with information on FP in Uganda. It is not surprising that men were more aware of condoms as an FP method than any other method while women were more aware of the longer-term FP methods. It may be argued that whereas men may be more focused on the dual protection they can get from condoms, women are usually more concerned about the prevention of unplanned pregnancy and this explains why men may be more informed more about condom use. These findings contradict those found in Mwanza region Tanzania [7], Mpigi district Uganda[6], Mbeya region Tanzania [11], and Nigeria [12] which all found that men had little knowledge on the subject.

Whereas females had more knowledge on FP and its availability within their communities, their attitudes, and fears about the possible side effects were significantly different from those of males. The study found out that more males were worried that modern FP methods can result in infertility while females were on the other hand more concerned about reduced sexual pleasure or their partners being angry if they found out. Whenever such fears exist, it becomes difficult for people to embrace FP methods. The mismatch in fears between men and women related to FP may also affect couples’ communication on FP matters and result in lower uptake of FP and or discontinuation. The negative effect of fear of side effects on FP uptake has been reported by previous research studies [6, 7, 13–16].

Findings revealed that half of the males that reported condom use as the current FP method also cited STIs/HIV prevention as the reason for its use. This finding revealed that most of the males preferred condom use dual protection compared to a third of the females resulting in a potential overestimation of reported FP use among males. Whereas almost half of the males in this study commonly reported condoms as their FP method of use, other studies have associated condom use with HIV prevention and protection efforts [17] or casual sexual relationships rather than as an FP method [18]. Future research should establish the primary use of condoms whenever they are reported as the FP method to avoid overstating its use as an FP method. The findings further suggested that the use of short-term modern FP methods accounted for more than three-quarters of the total modern FP methods in this study and recent studies associated short-term methods use with high odds of discontinuation and switching affecting uptake and retention of clients on FP [2, 16]. Our study findings from the regression model suggested that males, the young adults, the highly educated, and those in marriage or active relationship were more likely to use modern FP services. These findings are consistent with other research studies that reported high FP among the educated [8], and the married [19]. Contrary to the recent studies [8, 20] our study finding revealed no significant variations in uptake of modern FP among respondents with no child and those with an increasing number of children.

Results from a recent randomized controlled trial study found that where CHWs were active in the provision of low-cost health products and basic child health services to low-income families, there were improved health outcomes among the community members like reduced morbidity and mortality [21]. Our study findings revealed low involvement of CHWs in the delivery of family planning services. Learning from findings on the RCT study [21], we think that there is a potential opportunity for research on how CHWs can be leveraged to provide FP services and follow-up of clients. The Living model that addresses some of the bottlenecks to effective CHW services delivery like inadequate tools, lack of supervision, and compensation is a learning opportunity on how CHWs can be supported to drive impact in the community in the sexual and reproductive health services domain.

Our findings are not indicative of the national picture and should not be decisively used to generalize the entire country’s situation since they are largely biased to a few selected districts where Living Goods Uganda has a presence. Albeit, the findings provide important sex-disaggregated insights into the knowledge, attitudes, beliefs, and use of FP and are thus important for improving the programming of future FP interventions. The results showed that females had a higher positive attitude and beliefs on availability, affordability, and beliefs of FP methods than males. Overall both males reported high self-efficacy and this can be leveraged to promote use of FP methods. The study couldn’t succinctly establish the reasons for condom use or the differentiated effect of condom use on modern contraception as a dual protection method for STIs/HIV and pregnancy. Follow-up studies will aim to measure this accurately. Given that this study was conducted as part of a baseline to an FP project, project strategies and implementation will take into consideration the existing differences by sex as documented in this paper and devise sex-tailored approaches to improve FP knowledge, attitudes, and use in this population.

## Conclusions

Our study found out that knowledge of at least one modern FP method was high, but small significant differences were revealed between males (96 %) and females (98 %). Large differences were however observed in FP-specific methods. There was high self-efficacy about family planning methods use in both males and females. Significantly, more males (40 %) believed that modern FP methods can result in infertility compared to females (35 %). A significantly lower proportion of married females reported using or their partner using a modern FP method (39 %) compared to 45 % reported by the married males. The young adults and more educated respondents were more likely to use FP methods. Project strategies and implementation should take into consideration the observed sex differences in attitudes, beliefs, and knowledge and devise sex-tailored approaches to improve FP uptake. There was increased reporting of condom use as an FP and STI/HIV prevention method, follow-up studies aiming at succinctly measuring dual protection, and its drivers in both males and females should be done.

## Data Availability

Data and study tools are available upon request from the corresponding author.

## Abbreviations

AOR: Adjusted Odds Ratio
CHW(s): Community Health Worker(s)
CI: Confidence Interval
FGDs: Focus Group Discussion
FP: Family Planning
HH: Household
IUDs: Intra-Uterine Device
LG: Living Goods
MCH: Maternal and Child Health
MOH: Ministry of Health
MUREC: Mildmay Uganda Research and Ethics Committee
ODK: Open Data Kit
UBOS: Uganda Bureau of Statistics
UDHS: Uganda Demographic and Health Survey
UNCST: Uganda National Council for Science and Technology
